# Dynamic financial and monetary security risk assessment based on information service security assessment model and blockchain

**DOI:** 10.1038/s41598-023-45977-5

**Published:** 2023-10-31

**Authors:** Jia Li

**Affiliations:** Northwest University, Xi’an, 710127 China

**Keywords:** Computer science, Information technology, Scientific data

## Abstract

With the rapid development of blockchain technology, the financial and monetary (FM) blockchain fields also began to collide. Therefore, this study aims to explore the relationship between the two fields and efficiently evaluate the security of financial information. Firstly, this study introduces the theoretical basis of blockchain in the dynamic linkage mechanism of FM and gives the overall framework of digital currency based on blockchain. Meanwhile, the relationship between blockchain and digital finance is empirically analyzed and designed. Secondly, the framework of financial information service security assessment (ISSA) is created using blockchain technology, and the frame of security risk evaluation is verified by taking electronic invoice information as the research object. Finally, the results show that: (1) foreign exchange (forex) events and major stock index decline events have a significant impact on the return of Bitcoin (BTC) in the short term. Moreover, more than 70% of uncertainty events will make BTC’s abnormal return (AR) significantly positive; (2) Under the influence of forex uncertainty events, only one BTC’s AR is remarkably negative in short order, while the other seven times are markedly positive. In the uncertainty events of major stock indexes, only two times are significantly negative, and the other six times are positive. This indicates that uncertain events in the short run make prominent AR of BTC; the proposed blockchain-based ISSA model and assessment index are scientific, feasible, and operable.

## Introduction

Blockchain mainly comprises smart contracts and Ethereum (ETH)^1, 2^. The smart contract is a code and data collection stored in the ETH blockchain with the contract account address^3^. It is a “trigger” that can be automatically executed on the distributed database. It can automatically execute peer-to-peer transaction processing and data operation when the contract conditions are met^4, 5^. ETH improves the scalability of blockchain financial systems and lays a foundation for the birth of intelligent finance^6, 7^. At present, the research on the dynamic linkage mechanism of financial and monetary (FM) and security risk assessment based on blockchain is a topic of widespread concern in academic and industrial circles. With blockchain technology's continuous development and application, its role in the financial field is increasingly important. Blockchain technology, with its characteristics of decentralization and immutable information, provides strong support for applications such as digital currency (DC) and smart contracts. Besides, the financial field is closely related to blockchain technology, so the combination of blockchain technology and the financial field has become one of the hot spots in today's research.

However, with the advent of the digital age, traditional financial transactions face many challenges and problems, including low security and high-risk coefficients. These issues mainly stem from the centralized structure of traditional financial transactions and the reliance on third-party intermediaries. To address these issues and promote innovation and development in the financial industry, the main purpose of this study is to gain a deeper understanding of the relationship between blockchain technology and the financial market, and to provide a new trading model to overcome the problems of low security and high-risk coefficient in traditional financial transactions. Therefore, the research questions in this study are as follows. Firstly, the problems existing in traditional financial transactions are analyzed, including security and credibility challenges, and how these problems affect the stability and development of financial markets. Secondly, the application potential of blockchain technology in the financial field is explored, and how to improve the security and credibility of traditional financial transactions by introducing blockchain technology. Finally, the novelty of this study is introduced, involving the proposed security assessment model of financial information services and the in-depth study of the dynamic relationship between blockchain and the financial market, offering useful thinking and guidance for the future development of the financial industry.

Based on the above findings, this study explores the dynamic linkage mechanism of blockchain-based financial markets to promote the innovation and development of the financial industry. Through the introduction of blockchain technology, the study aims to improve the security and credibility of traditional financial transactions and provides a security risk assessment framework and an information service security assessment model to enhance the information security and credibility of the financial market. By studying the relationship between blockchain and financial markets, this study offers some thinking and guidance for the future development path of finance. This study hopes to support the innovation and digital transformation of financial markets, provide financial institutions, government regulators, and investors with a safer and more credible financial trading environment, and promote the sustainable development of the financial industry.

## Literature review

Many scholars have researched the application of blockchain in the FM field. Liu et al. studied the application prospect of smart contract automatic generation of blockchain in the financial field after multi-mode modeling^8^. Melo et al. further expanded the “debt-deflation” theory and comprehensively considered lenders in the analytical framework^9^. Pei and Li developed the relevant theory from the capital market perspective. He demonstrated the “Fire Sale” effect of debt liquidation from the two channels of monetary value and the wealth effect of asset price decline^10^. Abras et al. implemented a Financial Accelerator model according to the above theory. They developed a complete analytical framework for the self-reinforcing effect of deflation by combining information asymmetry theories and financial market imperfection^11^. To summarize, blockchain has two primary application paths in the FM fields. The first path was the development of original blockchain finance projects, represented by BTC, Ethereum, and available finance emerging in 2019^12^. The second path was “finance + blockchain”. In other words, financial regulatory authorities represented by Central Banks adopted blockchain to improve administrative and regulatory efficiency. In contrast, traditional financial institutions designated by commercial banks used blockchain to improve processes, reduce costs, and advance efficiency. Emerging technology enterprises, such as third-party payment companies, communication enterprises, and social media, extensively carried out financial business with blockchain^13^. Meng and Zhang examined the application of blockchain technology in financial markets, with a special focus on smart contracts and consensus algorithms. The study pointed out that blockchain could improve the security and transparency of financial transactions, but existing research mainly focused on technical details and ignored practical applications in financial markets^14^. Fisch et al. utilized smart contracts, consensus algorithms, and privacy-preserving technologies when researching the application of blockchain technology in the financial sector. They found that while blockchain could improve the efficiency and trustworthiness of financial transactions, there were performance and scalability challenges^15^. Zhao and Feng summarized the application and regulatory status of blockchain technology in financial services, and research showed that blockchain could enhance the efficiency and transparency of financial services, but regulation and laws and regulations were key issues^16^.

In summary, existing research has explored the application of blockchain technology in the financial market, but mainly focuses on the technical level, ignoring the practical application and performance scaling issues. In addition, regulatory challenges are overlooked. This study focuses on the impact of regulation and laws and regulations on the application of blockchain in financial services, aiming to offer a more comprehensive perspective and useful thinking for the innovation and development of the financial industry. Different from previous studies, this study focuses on the dynamic linkage mechanism of blockchain technology in financial markets and security risk assessment. While previous research has explored the application of blockchain in the financial field and related theories, this study aims to provide predictions of exchange rate fluctuation trends and support practical decision-making by modeling and analyzing the relationship between different currencies. The research path provides a more practical application direction for financial markets, emphasizing the potential value of blockchain technology in the financial field.

## The application of blockchain in the FM linkage mechanism

### The theoretical basis of blockchain in the application of the FM linkage mechanism

#### Alleviating information asymmetry

Growth and volatility are the most fundamental core issues in the FM fields. The solution of information asymmetry not only helps to reduce the volatility and systemic risks of the financial system but also improves the efficiency of actual economic activities and resource allocation and enables finance to empower the real economy^17^.

Thus, investigating each blockchain project as an independent system can alleviate information asymmetry. However, the interworking barriers between blockchain systems limit their application space, resulting in many “information islands”^18^. In traditional financial markets, the connectivity of human resources, commodities, and capital plays a vital role in expanding market boundaries, improving the efficiency of resource allocation, and enhancing market effectiveness^19, 20^. To solve this problem, cross-chain technology comes into being, making blockchain expected to become a new generation of valuable internet^21^.

#### Reducing the organization and transaction costs of the financial sector

**Figure 1:**
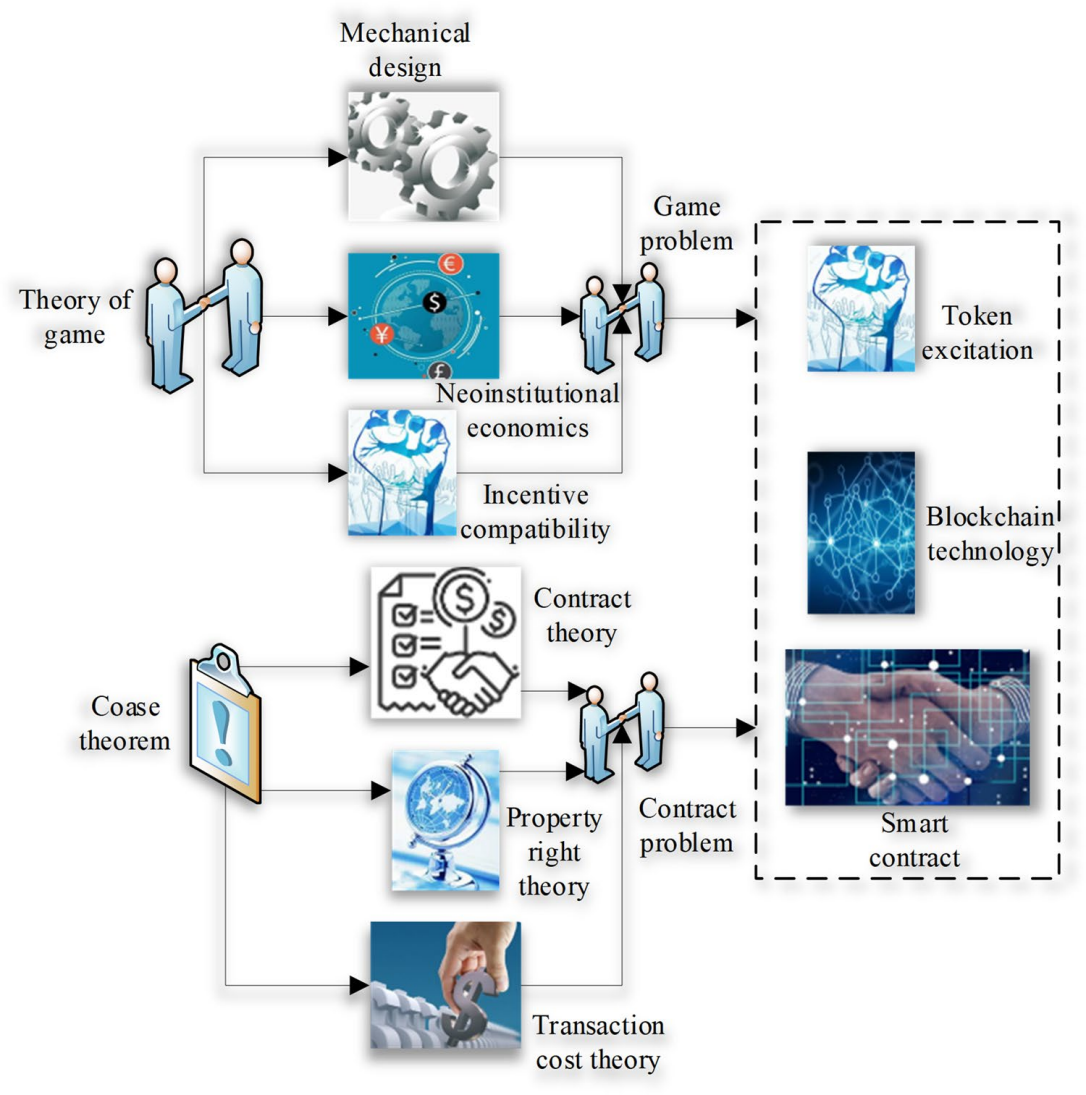
Theoretical basis of the reform of financial organization management mode.

Blockchain can also play a crucial part in reducing the organizational cost of enterprises^22^. Modern management has two core problems, one is the game, and the other is the contract^23^. Compared with artificial intelligence, cloud computing, edge computing, and other technologies that focus on improving productivity, blockchain, which changes how micro-individual information and value interflow, is expected to reshape production relations^24^. Blockchain has profoundly changed enterprises’ incentive models and governance mechanisms, causing the re-matching of management elements. Token governance provides solutions to game problems along the path of “game theory—mechanism design—new institutional economics—incentive compatibility”. Smart contracts offer solutions to contract problems along the path of “Coase theorem—contract theory—property rights theory—transaction cost theory”, as denoted in Fig. 1^25, 26^.

#### Forming a more complete financial network

The blockchain economic model is a network economy closer to the idealized state^27^. The network economy is a structure, not necessarily based on the internet. The trading and the monetary systems are also network economies^28^. Daria and Alessandra summarized seven characteristics of the network economy during the swift growth of the internet economy: unlimited time, globalization, virtualization, a direct connection between production and marketing, coexisting competition and cooperation, high efficiency, and innovative economies^29^. Seven characteristics of the internet economy have been further developed in the digital financial market based on blockchain. DC's “7 × 24 × 365” market transaction mechanism has truly realized the economy without a time limit; DC broke the boundary between countries and achieved global pricing and trading^30^.

### Blockchain and DC

#### The relationship between blockchain and DC

**Figure 2:**
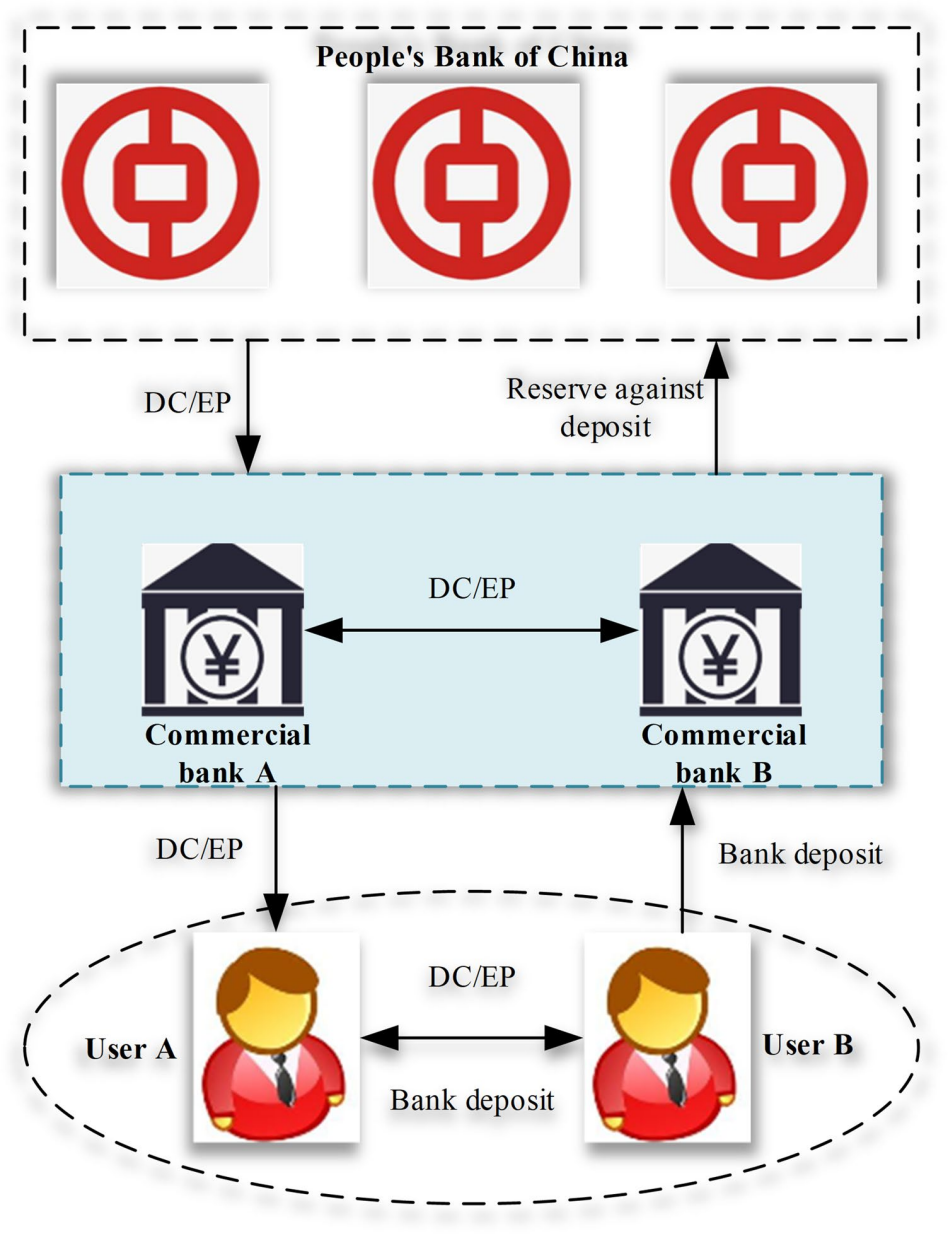
The binary framework of “Central Bank-CB” in DC/EP.

In fact, legal DC is not necessarily based on blockchain. In promoting legal DC, the People's Bank of China (PBC) needs to preset the technical route. Because if the direction of legal DC technology is wrong, the loss is systemic. Thereupon, the PBC only explicitly provided the policy objectives and overall framework of DC/ Electronic Payment (EP), while keeping an open attitude towards the technical route to achieve these objectives^31, 32^. The binary framework of “Central Bank-commercial bank (CB)” in DC/EP is presented in Fig. 2.

Figure 2 displays that DC/EP is essentially an encrypted string guaranteed by the Central Bank and issued by signature. Its role is to replace cash M0, hold DC/EP without interest, and issue and circulate following the existing binary framework of “Central Bank-CB”. The framework is universal and ubiquitous. It does not rely on specific transaction mediums and payment channels or assume other functions except the four basic functions of circulation means, monetary value measure, payment means, and value storage.

#### The overall framework of DC

**Figure 3:**
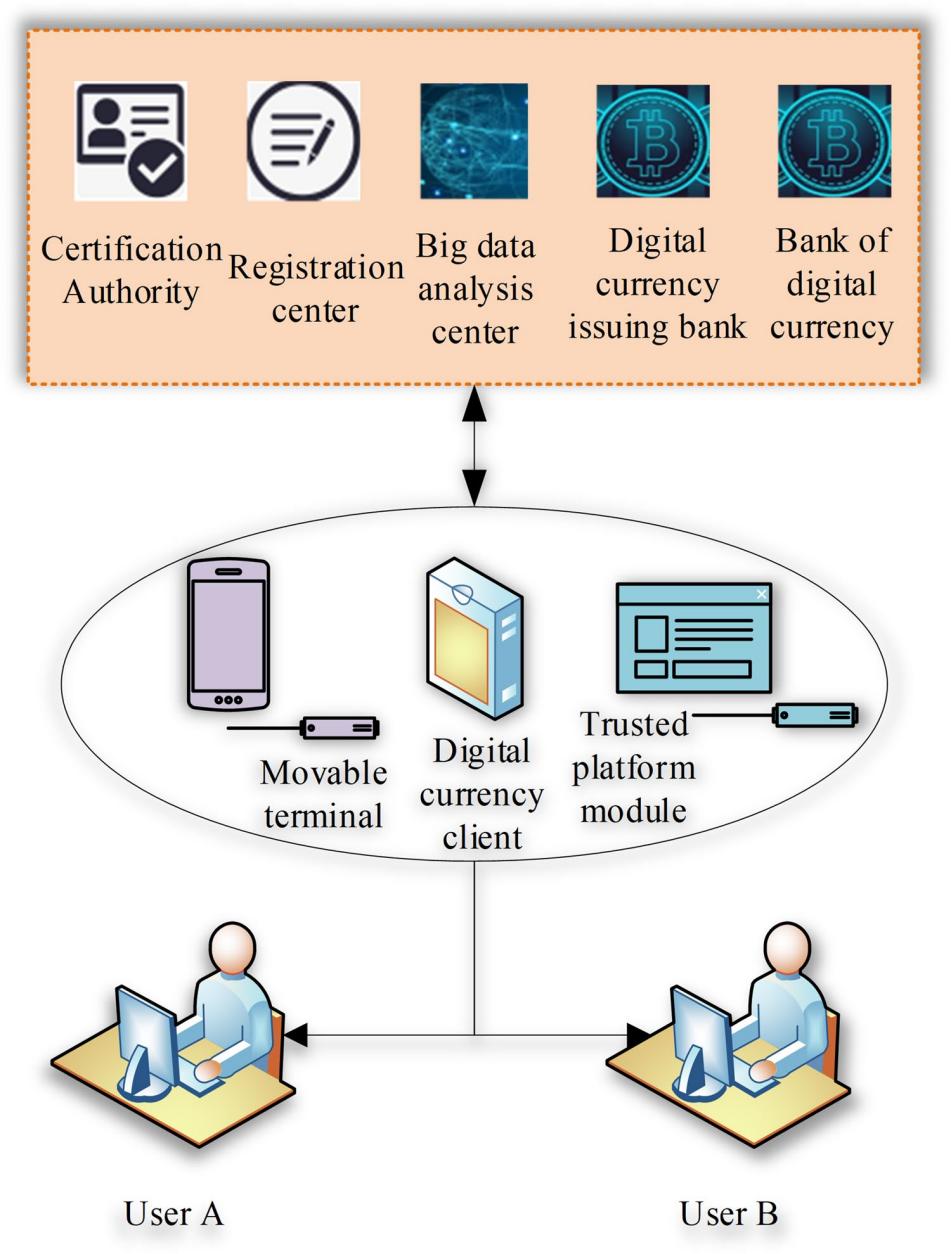
The framework of “one currency, two libraries, and three centers”.

China's legal DC is still in the research and internal testing stage. According to the statements of relevant central bank personnel, DC/EP adopts the binary investment system of “Central Bank-CB” and the operating framework of “one currency, two libraries, and three centers”. The framework structure is displayed in Fig. 3.

Figure 3 describes that “one currency” refers to the DC/EP token guaranteed by the Central Bank, and “two libraries” stand for the issuing bank of the Central Bank and the bank treasury of the CB, which reflects the characteristics of the binary investment system. DC/EP is first transferred between the Central Bank and CB, that is, the issuance and withdrawal of DC/EP, and then moved from a CB to residents and enterprises. The “three centers” are the technical support for DC/EP distribution and circulation, including the registration, certification, and big data analysis centers.

### Blockchain and digital finance

To explore the relationship between blockchain and digital financial assets, the Event Study Methodology (ESM) and the Value At Risk-Generalized Autoregressive Conditional Heteroskedasticity (VAR-GARCH) model are employed to analyze the hedging properties of digital assets (DAs) represented by BTC in the short and medium and long term. ESM is a quantitative method used to analyze the impact of specific events on asset prices and markets, often used for short-term event analysis. The VAR-GARCH model is a statistical model employed to measure and predict asset price volatility and is often utilized to study long-term and short-term risks. The application of these two methods in this study is to explore the hedging properties of DAs in the short, medium, and long term. ESM is applied to analyze the short-term price impact of a particular event on DAs, while the VAR-GARCH model measures the price volatility of DAs to understand their long and short-term risks. Applying these two methods in this study provides insight into the hedging properties of DAs, particularly their performance in macroeconomic and financial markets.

In the analysis of short-term and medium-long-term effects, the sources of risk are divided into macroeconomic risk and financial market risk, and different variables are used as proxies to clarify the real sources of risk; By reasonably selecting the empirical data span, this study compares and analyzes the hedging ability of gold and BTC, which is known as “digital gold”, and examines the dynamic and time-varying relationship between them; The hedging of BTC is investigated from two dimensions of price and trading volume for the specific holder structure of BTC.

#### A study on the short-term effect of DA hedging

The data presented in the table below is derived from Coin Market Cap, which not only counts information about different DA markets (including market capitalization, price, and circulation of different DAs), but also provides extensive DA market data. In addition, this data is provided to researchers and developers in the form of an Application Programming Interface (API) so that they can access and analyze this information. Taking April 19, 2022, as the deadline, the types, issuing countries, and market value of crypto DAs worldwide have been counted, and the world’s top 5 crypto DAs have made statistics. The statistical results are exhibited in Tables 1 and 2.

**Table 1:** Statistics of crypto DAs worldwide.

The types of crypto DAs	1730
Total market value of crypto DAs	$350,912,204,383.3
The sovereign country where the issuing entity is located	These are mainly concentrated in China, the United States, Britain, Australia, France, Japan, and others
The proportion of TOP5 crypto DAs in market value	70.22%
The proportion of TOP5 crypto DAs in market value	77.88%
The proportion of TOP5 crypto DAs in market value	82.49%

**Table 2:** Statistics of TOP5 crypto DAs in market value.

Name	Abbreviation	Market value	Price	Circulation	Proportion (%)
Bitcoin	BTC	$139,763,368,381	$8227.60	16,987,137	39.8286
Ethereum	ETH	$53,961,163,088	$545.51	98,918,380	15.3774
Ripple	XRP	$28,614,177,503	$0.73	39,122,794,968	8.1542
BTC Cash	BCH	$16,128,597,987	$944.18	17,082,213	4.5962
Litecoin	LTC	$7,955,869,681	$141.68	56,154,588	2.2672

Additionally, short-term hedging examines the nature of hedge funds entering the market when the external political and economic environment suddenly has a relatively drastic change. Thereby, the transaction price of BTC in the sample period is selected as the research variable, and the ESM is utilized to investigate the short-term hedging effect of DAs under the uncertainty of macroeconomic and financial markets.

##### Event definition and selection criteria

The selected specific events are divided into foreign exchange (forex) and stock market uncertain events used as risk proxy events from macroeconomic and financial markets. The number of event samples and the specific selection criteria are plotted in Table 3.

**Table 3:** Selection criteria and sample quantity of events.

Type of events	Selection and criteria	Sample quantity of events
Forex uncertain events	Through collecting events in the time sample interval of public data, the impact of the events on the forex rates of major currencies (US dollar (USD), Euro, RMB, Canadian dollar (CAD), Swiss franc (CHF), etc.) is analyzed, and the negative events are selected	8
Stock market uncertain events	The benchmark measure of the Dow Jones Index, representing the global stock market, is selected with 1.5% as the standard (combining the events of consecutive declines in adjacent trading days)	8

According to Table 3, specific events are screened in this study, as outlined in Tables 4 and 5.

**Table 4:** Financial events in the international forex market.

Serial number	International forex market uncertainty event	BTC price (USD)	The rising range of BTC (%)
1	The Swiss National Bank (SNB) announced that it would abandon the lower limit of EUR/CHF 1.20, and many forex platforms were affected by this event, resulting in varying losses	209.8	0.2720
2	The PBC declared a one-off devaluation of nearly 2% of the RMB's midpoint rate. The onshore RMB depreciated 1.87% against the USD	269	0.0218
3	The British pound fell sharply after Brexit passed the referendum	656.9	0.0581
4	The UK Financial Conduct Authority reduced the trading leverage to 50:1, causing industry shock	758.2	0.0051
5	Forex Capital Markets (FXCM), the largest retail forex broker in the US, was forced out of the market	1049.60	0.0243
6	FXCM was removed, and the board of directors was all shuffled	1119.00	0.0385
7	The USD fell against the CAD after the Bank of Canada (BOC) raised interest rates	2403.10	2403.10
8	The BOC raised interest rates from 0.75 to 1%. The USD fell against the CAD	4618.70	0.0475

**Table 5:** Financial events in the stock market and their index values.

Sequence number	Stock index	The range of rise and fall (%)
1	17,501.65	− 1.86
2	17,387.21	− 1.65
3	17,856.78	− 1.54
4	17,662.94	− 1.85
5	17,718.54	− 1.62
6	17,826.30	− 1.54
7	17,596.35	− 1.95
8	16,990.69	− 2.06

##### Estimation of normal return (NR) and abnormal return (AR) of BTC

For the estimation of AR and NR of BTC, the NR model selected in this study is the mean adjustment model, as indicated in Eqs. (1) and (2):

1 $$ER_{t} = \frac{1}{k}\mathop \sum \limits_{{t = T_{0} + 1}}^{T1} R_{t}$$

2 $$AR_{t} = R_{{t_{ev} }} - \frac{1}{k}\mathop \sum \limits_{{t = T_{0} + 1}}^{{T_{1} }} R_{{t_{es} }}$$

$ER_{t}$ indicates the estimated NR of BTC during the event window; $R_{t}$ stands for the actual return value of BTC in the estimated period; $AR_{t}$ and $R_{{t_{ev} }}$ represent the abnormal and actual return during the event window; $R_{{t_{es} }}$ means the actual return of the estimated window period, and k refers to the length of the event estimation window. The length of the estimation window selected here is 240 days. At the same time, the ESM adopted mainly aims at the short-term effects of events on currency earnings. Thus, the event window period is $T1 + 3$.

##### AR test

Generally speaking, the statistical test of AR is divided into parametric and non-parametric estimations. This study selects the former to test AR. The parameter estimation statistic ***S***_***t***_ of AR is constructed, and its specific form is as follows:

3 $$S_{t} = \frac{{\frac{{AR_{t} }}{{\sqrt {var(AR_{t} )} }}}}{{\sqrt {\frac{k - 2}{{k - 4}}} }}$$

The original assumption of S is that the AR is zero within the event window, and this statistic is subject to standard normal distribution.

#### Empirical design

To explore the relationship between blockchain and DAs and determine the short-term impact of blockchain technology on digital asset prices and market fluctuations, this study proposes the following empirical design. Firstly, DA prices, forex uncertainty events, stock index uncertainty events, and supply and demand data on the DA market are collected. Next, the short-term impact of forex and stock index uncertain events on BTC returns is analyzed through the AR model, and its significance is tested. Moreover, the AR model of BTC returns is calculated and tested for significance. Finally, the experimental results are analyzed, focusing on the impact of forex and stock index uncertainty events on BTC, and whether blockchain technology shows significant gains as a hedging asset in times of uncertain events.

## The blockchain-based information service security risk in the domain of FM

In the previous section, the relationship between blockchain and FM is analyzed. This section will employ blockchain technology to evaluate and study FM’s information service security risks.

### The general process of ISSA

**Figure 4:**
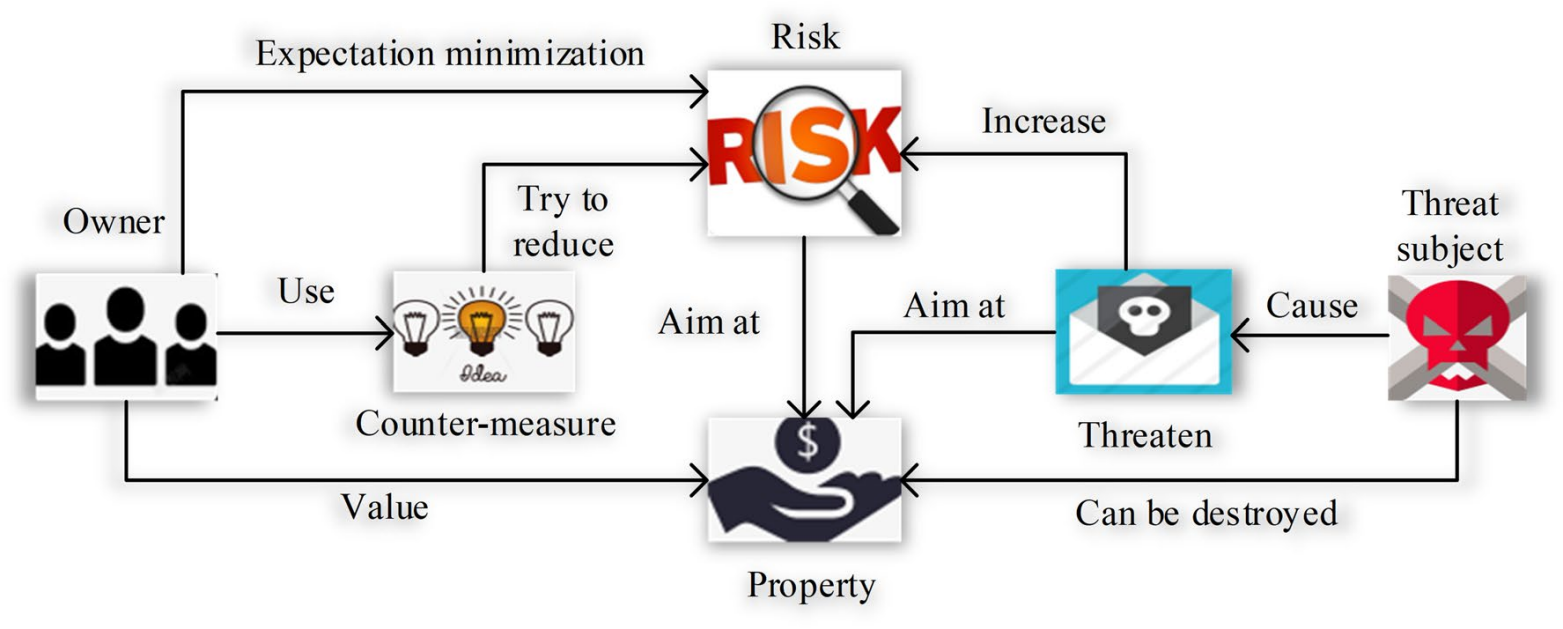
General procedure of ISSA.

The common workflow of ISSA is shown in Fig. 4.

Figure 4 signifies that the information system owner reduces risk to protect tangible and intangible assets by using a variety of security controls, including personnel, technology, engineering, and management; The actual or supposed threat subject wishes to abuse or destroy the asset; The owner will realize that this threat may cause the asset to be damaged and the value of the asset to the owner will be reduced; The owner seeks to minimize risk by adopting security policies and imposing other constraints.

### Blockchain-based ISSA model and security requirements

#### Security objectives from the perspective of information lifecycle management (ILM)

Security objectives are further expanded in each stage of ILM. The proposed Target of Evaluation (TOE) security objective and traceability of safety issues are detailed in Table 6.

**Table 6:** Security objectives and safety issues of TOE.

Safety issues			Security objectives
Security threat	Data risk	Data leakage	Information generation
		Data forgery	
		Data tampering	Information processing
	Content risk	Illegal content	
		Distort content	Information delivery
		Vulgar content	
Organizational strategy	Regulatory requirements	Identity concealment	Information dissemination
		Contract concealment	
		Information concealment	Information storage
Other risks	Technical risk	Cryptographic security	
		Consensus mechanism security	Information destruction
		Smart contract security	

#### Blockchain-based ISSA model

**Figure 5:**
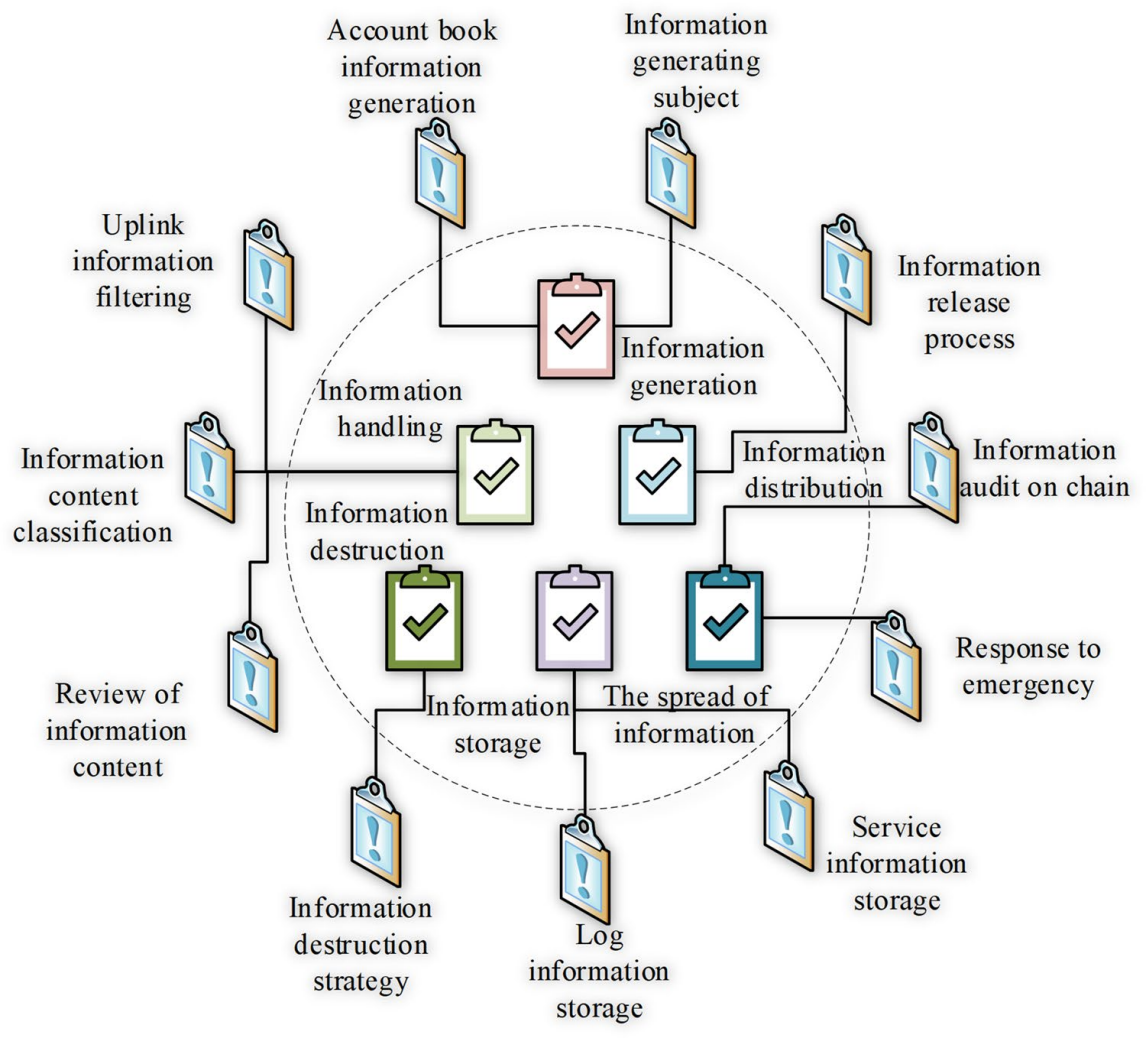
Information service security requirement model based on blockchain.

To facilitate all users, developers, and evaluators of guarantee work of blockchain information service security, a general blockchain-based ISSA model is put forward from three aspects: product type, ILM, and security requirements, guided by the idea of security engineering. The model is revealed in Fig. 5.

Figure 5 demonstrates that blockchain information service providers should first complete the platform security guarantee to ensure that platform deployment, resource scheduling, and user access meet relevant national requirements. Then, they need to focus on the security of blockchain information content; The user registration, information audit, information linkage, and anonymous release function are taken as the enterprise security risk prevention and management focus. Meanwhile, it is required to have the technical ability of information content security supervision. These providers should provide users and third-party evaluation agencies with basic information such as product category, service scope, user scale, adopted incentive mechanism, encryption mechanism, smart contract version information, operating system, current block height, etc.

Furthermore, corresponding to ILM, the security requirements of blockchain information content mainly cover identity authentication and real-name detection related to user identity; Pre-chain detection and on-chain supervision of information related to information content; And the rules and regulations, site environment, and other requirements related to safety assurance.

### Design of blockchain-based ISSA

The blockchain-based ISSA is designed to ensure the security and credibility of blockchain information services, and its creation and implementation include the following key elements:

#### The index system of blockchain-based ISSA

First, it is necessary to establish a comprehensive index system for evaluating various aspects of blockchain information services. The index system is divided into basic and enhanced levels, covering key areas such as information generation, processing, transmission, dissemination, storage, and destruction. For example, information generation includes the encryption, credibility, and integrity of information, while information transmission covers the evaluation of information content review and publishing processes. Considering the different influences and user scales of blockchain information services, assessment indexes can be divided into a basic level, and an enhanced level, and both are in a progressive relationship. The specific evaluation results can be “conforming” or “non-conforming”, “partially conforming” or “partially nonconforming”. The key design contents of each index are portrayed in Table 7.

**Table 7:** Content of index system of blockchain-based ISSA.

First-level indexes	Content of second-level indexes
Information generation	Information encryption, information trust, information integrity
Information processing	On-chain information filtering
	Information content classification
Information delivery	Information content review
	Information release process
Information dissemination	On-chain information review
	Emergency response disposal
Information storage	Business information storage
	Log information storage
Information destruction	Information destruction strategy

#### Experimental design of blockchain-based ISSA

Experiments need to be designed to verify the effectiveness of blockchain and the actual effectiveness of the ISSA index system. A suitable evaluation object is selected, such as the blockchain electronic invoice platform, to test the performance of ISSA. The experiment covers multiple aspects, including information generation, processing, transmission, dissemination, storage, and destruction, to evaluate the effectiveness of blockchain technology in improving information security and credibility. Based on this, the experimental design of the blockchain-based ISSA model to be validated in this study is as follows:

##### Collection of datasets

Datasets related to the security assessment of the blockchain electronic invoice platform are collected. These datasets include account authentication, account freezing and cancellation, account traceability, ledger information encryption, trustworthiness, and integrity. It also covers information content identification, filtering, and classification, audit standard management, audit procedure management, information release process, on-chain information security review, information security monitoring and early warning, security event grading plan, personal information storage, transaction information storage, log storage content, log hierarchical storage, information destruction strategy, etc.

##### Data analysis

The collected data will be processed and statistically analyzed, and the evaluation indicators of the blockchain electronic invoice platform will be evaluated.

The selected evaluation object is a blockchain electronic invoice platform. The reason for choosing the electronic invoice platform for the experiment is that it represents a field with strong practical application needs, and its security and credibility are crucial for financial and commercial transactions. By applying blockchain-based information security and credibility assessment in this field, the potential of blockchain technology in solving practical problems, improving information authenticity, and preventing tampering can be effectively demonstrated. In addition, the electronic invoice platform has a multi-stage data flow and processing process, consistent with blockchain technology's core characteristics. Therefore, choosing it helps demonstrate blockchain's advantages in information management and security. Most importantly, this choice provides important validation and practical demonstration for the blockchain-based ISSA, helping to promote the wider application of blockchain technology in the financial and commercial fields. The proposal of blockchain electronic invoices is mainly to solve the problems of multiple declarations of one vote, false declarations and false deductions, and difficulty verifying the true and false. Hence, each link can be traced, the information cannot be tampered with, and the data will not be lost. In addition, the evaluation system designed in the above section will still be selected to verify the effect of blockchain-based ISSA.

Furthermore, key subsequent steps in implementing the blockchain-based ISSA model include result analysis and feedback, implementation and monitoring, as well as documentation and reporting. First, in the result analysis and feedback stage, the information service layer that needs to be improved is identified by carefully analyzing the experimental results to enhance its security and credibility. Second, specific improvement strategies and recommendations are developed based on the evaluation results to ensure that information services reach a higher level with the support of blockchain technology. Then, improvements need to be implemented during the implementation and monitoring, and the performance of information services is continuously monitored. This includes conducting regular security reviews, monitoring, and early warnings, as well as implementing information destruction strategies to ensure the continued effectiveness of information security and credibility assessments. Lastly, in terms of documentation and reporting, the entire ISSA design, creation, and implementation process is thoroughly documented, generating professional reports for communication and sharing of experience with external parties. These reports will help other organizations and entities learn from and adopt similar blockchain-based information security and trustworthiness assessment methodologies to improve the quality and trustworthiness of information services jointly.

## Experimental results and analysis

### Analysis of the dynamic relationship of FM based on blockchain

#### The relationship between blockchain and digital financial assets

**Figure 6:**
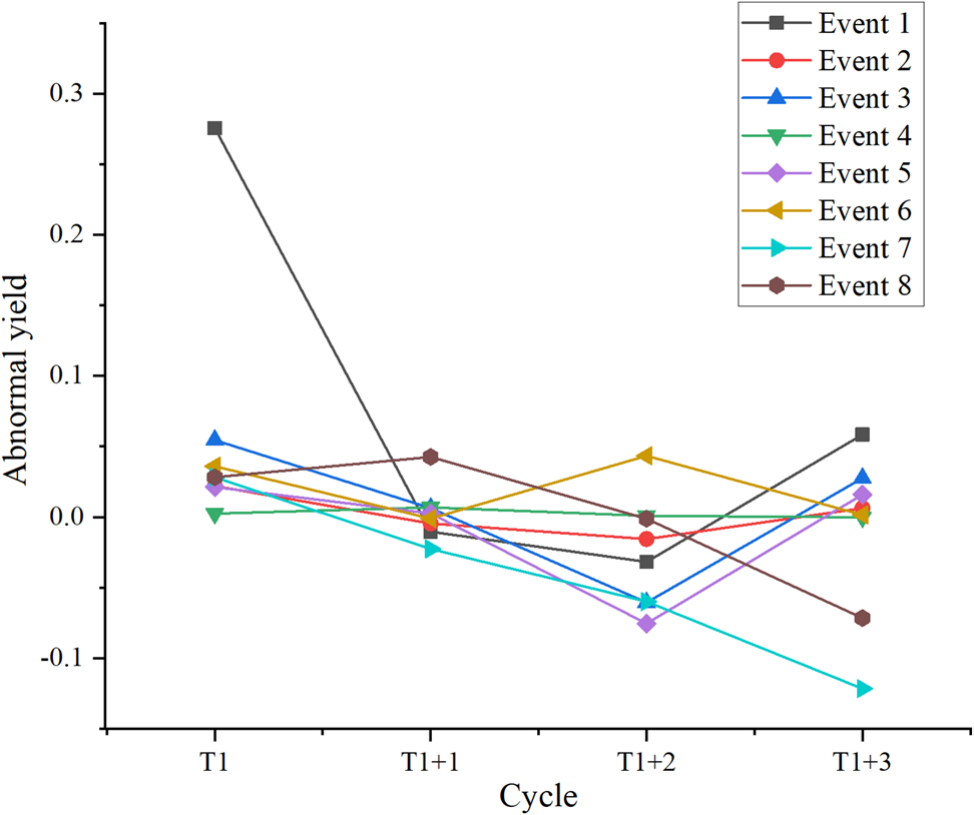
Abnormal impact of forex uncertainty events on BTC returns.

**Figure 7:**
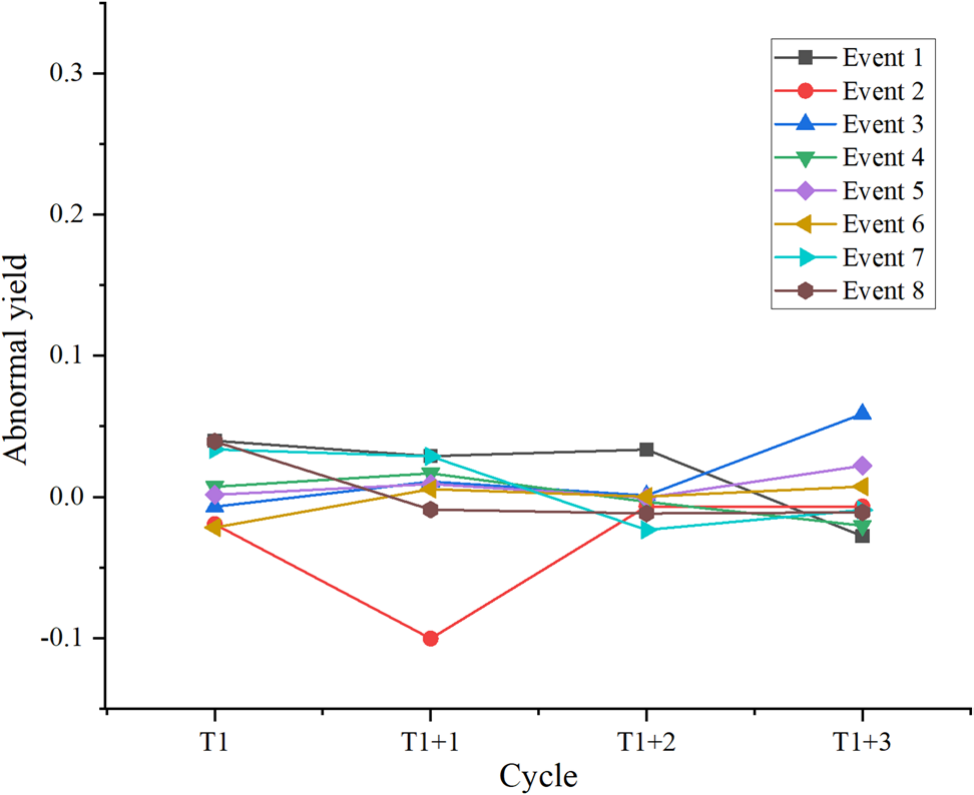
Abnormal impact of stock market uncertainty events on BTC returns.

According to the event definition, NR estimate, AR estimate, and significance test statistics of AR listed above, this section calculates the abnormal impact of uncertain events of forex and the stock market on the BTC yield. Moreover, the statistical value of the significance test of AR is listed. The specific results are suggested in Figs. 6 and 7.

Figures 6 and 7 denote that in forex uncertainty events, although only one AR of BTC is significantly negative, the other 7 times are notably positive in the short term. In major stock index uncertainty events, only 2 are obviously negative, and the other 6 are markedly positive. This means that uncertain events in the short term cause significant AR of BTC, and over 70% of uncertain events make the AR of BTC prominently positive. The above findings indicate that the fundamental causal relationship and mechanism of forex uncertainty events and major stock index decline events significantly impact BTC returns lies in the risk aversion triggered by uncertain events. When macroeconomic and financial markets face uncertainty, investors seek safe assets and shift their funds to the BTC market, expanding the demand for BTC and driving up the market price of BTC, resulting in a significant positive autoregressive AR effect. In this case, as a DA, BTC acts as a safe-haven asset in the short term, and its price has a sensitive positive response to market uncertainty. Therefore, BTC with blockchain technology shows a certain hedging effect, further emphasizing DAs' value in uncertain events and their role in financial markets.

In summary, a dynamic relationship between blockchain and FM promotes and restricts each other. As a typical representative of blockchain technology, the price of BTC is affected by many factors, including market demand, market supply, and traditional monetary policy. However, when the economy or financial markets face uncertainty, BTC as a safe-haven asset can show significant AR in the short term, this is undeniable. In addition, the above research results also show that blockchain technology has an important hedging effect in the context of uncertain economy and financial market, attracting safe-haven assets into the BTC market, pushing up the price and producing a significant autoregressive effect. In summary, BTC, as a safe-haven asset, presents significant hedging potential under uncertain events, further emphasizing blockchain technology’s important role in financial markets and providing important insights and strategic suggestions for financial decision-making on how to deal with uncertain events.

### Results of blockchain-based ISSA

On account of the available external research materials and verification results, the assessment results are outlined in Table 8.

**Table 8:** Security assessment results of blockchain electronic invoice platform.

Assessment indexes	Serial number	Specific assessment item	Result recording
Ledger information	1	Encrypt ledger information	✓
	2	Trusted ledger information	✓
	3	Complete ledger information	✓
Account of the platform	4	Account authentication of the blockchain platform	✓
	5	Account freezing and cancellation of the blockchain platform	✓
	6	Account traceability of the blockchain platform	✓
Information content	7	Identification and filtering of information content	✓
	8	Classification of information content	✓
	9	Authorization management of information content	×
Information review and release	10	Audit standard management	✓
	11	Audit procedure management	✓
	12	Information release process	✓
Information security	13	On-chain information security audit	×
	14	Information security monitoring and warning	×
	15	Safety incident classification plan	✓
	16	Emergency response technology	×
Information storage and destruction	17	Personal information storage	✓
	18	Transaction information storage	✓
	19	Log storage content	✓
	20	Log hierarchical storage	✓
	21	Information destruction strategy	×

✓ indicates that the relevant measures have been proved to meet the assessment indexes by the existing materials; × refers to non-conformance.

Table 8 reveals that the platform users are relatively controllable due to the limited number of alliance members, certification, approval, control, and authority configuration. The information content has an audit filtering mechanism and technical means pre-chain, and the risk of illegal information content being uploaded to the chain is limited. Although the monitoring mechanism and capability after the information is put on the chain need to be improved, it has specific emergency response and processing capabilities after the information security incident, and the risk of spreading wrong information is relatively controllable.

## Discussion

Compared to previous studies, this study has significant differences and more focus. Firstly, it focuses on the dynamic linkage mechanism and security risk assessment of blockchain technology in the financial market, which is different from previous research and mainly focuses on the application and theory of blockchain in the financial field. Secondly, the specific application scenarios of blockchain technology in the financial market have been deeply studied, especially in terms of currency exchange rates, offering predictions of exchange rate fluctuation trends by modeling and analyzing the relationship between different currencies, which is closely related to previous statistics. The study also highlights security risk assessment, which helps financial markets better understand potential risks and take appropriate preventive measures, which is also relevant to previous statistical analysis. Finally, it is more practice-oriented, highlighting the practical application potential of blockchain technology in financial regulation and process improvement, providing more details and in-depth discussions for financial markets, which can be directly related to previous statistics. In addition, based on statistics related to current financial events, the main forms of cooperation between current financial regulation and blockchain technology are studied, and the impact of these forms on market fluctuations and financial innovation. This further enhances the study's relevance and made the discussion more in-depth and relevant. In summary, as technology continues to mature and improve, blockchain technology is believed to promote innovation and development in China's financial sector. Therefore, for financial institutions and investors, mastering the development trends and security assessment methods of cryptocurrency and blockchain technology will help them better grasp market opportunities and risks, which is also significantly related to previous research.

## Conclusion

As the core driving force of DC, blockchain technology plays a crucial role in developing digital finance and DC. Therefore, this study aims to explore the relationship between blockchain and financial markets and the potential applications of blockchain in the financial field. First, from theoretical and empirical perspectives, in-depth research is conducted on the relationship between blockchain and legal DC and between blockchain and digital financial assets. Legal DC, as a typical representative of the “finance + blockchain” path, has been found to involve central banks and CB, providing a new perspective for financial regulation and monetary policy. The DA market represented by BTC has a certain hedging effect on macroeconomic and financial markets in the short term, which is significant for risk management in financial markets. In addition, it is observed that there is a mutual constraint and promotion relationship between the dynamic mechanisms of the financial market and blockchain, indicating that blockchain technology has a positive impact on the development of the financial market. Second, the ISSA model is designed and implemented based on blockchain technology and information service security theory. By verifying the model's effectiveness, it is concluded that it has relative controllability in generating, processing, disseminating, and storing risks of adverse information in the evaluation object, which is conducive to improving the security and credibility of financial information services. This has played a positive role in information flow and risk management in the financial market.

However, it should be noted that this study also has some limitations. Firstly, blockchain research in the financial market field is still relatively preliminary and has not yet covered all potential application scenarios and problems. Secondly, the research results are mainly based on theories and models and have not been widely verified in the financial market. Hence, future research directions can include a wider range of financial market entities, further in-depth analysis of the application of blockchain technology in the financial field, and more empirical research to verify the actual effectiveness of theoretical models. In addition, in the future, more research directions will be explored, such as the application of blockchain technology in financial market regulation and compliance, as well as the relationship between blockchain and different types of financial assets. Moreover, with the continuous development and maturity of blockchain technology, attention will be paid to its further application in the era of financial innovation and digital economy to promote the development and security of the financial market. In conclusion, this study provides a preliminary understanding and framework for the relationship between blockchain and financial markets, providing useful references for future research and practice.

### Supplementary Information

Supplementary Information.

## Data Availability

All data generated or analysed during this study are included in this published article (and its Supplementary Information files).
